# Stress and Anxiety among High School Adolescents: Correlations between Physiological and Psychological Indicators in a Longitudinal Follow-Up Study

**DOI:** 10.3390/children10091548

**Published:** 2023-09-14

**Authors:** Gábor Pál Stromájer, Melinda Csima, Réka Iváncsik, Bernadett Varga, Krisztina Takács, Tímea Stromájer-Rácz

**Affiliations:** 1 Institute of Basics of Health Sciences, Midwifery and Health Visiting, Faculty of Health Sciences, University of Pécs, H-7621 Pécs, Hungary; gabor.stromajer@freemail.hu (G.P.S.); bernadett.varga@etk.pte.hu (B.V.); krisztina.takacs@etk.pte.hu (K.T.); 2 Institute of Education, Hungarian University of Agriculture and Life Sciences, H-7400 Kaposvár, Hungary; petone.csima.melinda@uni-mate.hu (M.C.); ivancsik.reka@uni-mate.hu (R.I.); 3 Institute of Diagnostic, Faculty of Health Sciences, University of Pécs, H-7621 Pécs, Hungary

**Keywords:** adolescent, stress, anxiety, cortisol, school

## Abstract

Mental and psychological disorders are serious health problems worldwide. Anxiety among high school students can affect school performance, relationships, and family life. Objective: Our aim is to understand the anxiety levels and associated factors among high school students and compare the results of psychological tests measuring anxiety with the cortisol levels obtained from biological sampling. Method: In our longitudinal follow-up study, we involved 125 individuals in May 2019. Validated measurement tools were used during questionnaire data collection, including the State–Trait Anxiety Inventory, Clear Communication Scale, Multiple Social Perceived Support Scale, and related HBSC questions. As objective data, we collected hair samples for cortisol level measurement. Results: At the end of the school year, the anxiety levels measured by psychological tests were significantly higher (*p* = 0.001) compared to the anxiety levels at the beginning of the next school year. Anxiety levels were higher among girls and were influenced by the type of school and parental expectations. Both state anxiety and trait anxiety showed a strong correlation with psychosomatic symptoms (*p* < 0.001) and anxiety arising from school expectations (*p* < 0.05). The changes in cortisol levels did not follow the changes in psychological tests. Cortisol level increased (*p* = 0.01) in the second sample.

## 1. Introduction

Stress is a biological phenomenon that is inherent to life, but it can also be a precursor to various diseases. Stress is the description of the collective non-specific reactions of the organism in response to any kind of circumstances that disturb its equilibrium. Stressors can be external or internal factors, or according to another classification, environmental, biological, and mental factors. Free radicals are produced as a result of stress, which can damage cells when the internal balance, homeostasis, is disrupted and the quantity of antioxidants decreases. The overabundance of free radicals can lead to oxidative stress, which can damage lipids, proteins, tissues, and DNA, resulting in molecular changes that later contribute to the development of diseases [1,2]. It has been proven that both acute and chronic stress significantly contribute to the development of cardiovascular diseases [3]. Acute mental stress results in increased heart rate and elevated blood pressure through the release of catecholamines. Chronic psychological stress exposure can lead to the development of conditions such as atherosclerosis, hypertension, and metabolic disorders [4]. When psychological stress is accompanied by physical stress, it can worsen cardiovascular reactions, collectively contributing to the development of cardiovascular diseases and increasing mortality rates [5]. Chronic stress also affects the immune system, making the body more susceptible to infections due to excessive worry and anxiety [6]. In addition to its impact on physical health, stress can also influence cognition, social relationships, and work and school performance. Given the multifaceted effects of stress, it is of paramount importance to address it at both individual and community levels [7].

The literature distinguishes between two major categories of stress: beneficial or eustress, and distress, which results in negative stress experiences. Eustress always has a positive impact. Among animals, it aids in survival, escape, and prey acquisition. In humans, eustress is a short-lived experience that focuses efforts, activates reserves, and provides motivation. It can manifest in situations such as exams, emergencies, or even in the case of extreme athletes [8]. Eustress triggers numerous physiological processes that prepare the body for a response within a short period of time.

Distress is present in an individual’s life as a negative stress experience. The body is continuously exposed to the effects of stress, with stressors persistently present, and the stress response is not recognizable. Distress is always harmful and unpleasant, and individuals experiencing stress have difficulty or are unable to cope with it. Regarding school-related stress, the role of stressors stemming from family and parental expectations, teacher expectations, or peer pressure is significant. Chronic stress during the prepubertal period can affect the hypothalamic–pituitary–adrenal (HPA) axis, leading to eating disorders [9], which can also be a possible “trigger” for unhealthy eating habits, with long-term consequences on childhood obesity [10] that can persist into adulthood. It is crucial to address the stress induced by the school environment, particularly for school-going children [11]. When the school environment functions as a stressor, it can result in long-term reduced self-esteem and constant anxiety among students [12,13,14]. It is a well-known fact that 75% of the causes of human diseases can be traced back to childhood [15], which is why anxiety prevention programs integrated into the school curriculum and implemented within the local school environment are of paramount importance. They can effectively reduce the level of anxiety and increase children’s access to mental health care services [16].

Numerous publications highlight that this is an increasingly common issue among high school students, alongside depression [17,18,19]. This is particularly significant for adolescents and young people because anxiety restricts their school abilities, affects their social relationships and family life, and often leads to severe problems such as eating disorders, depression, or even suicide. The last two years of high school, which involve preparations for higher education entrance exams, are a period of intense anxiety, stress, and worry for young students, which is extremely burdensome [20,21]. Over the past decade, based on numerous studies, it has been sufficiently proven that anxiety is associated with lower school performance [22,23,24,25,26,27,28], and it has a negative impact on successful admissions and career choices [29,30,31,32,33]. Anxiety disorders affect 10% of children up to the age of 16. They significantly impair daily functioning, often persist into adulthood, and increase the risk of other psychiatric disorders during adolescence and young adulthood [34,35,36,37,38].

### Cortisol as a Biological Marker

The assessment of anxiety and stress exposure is most commonly completed through questionnaire surveys (subjective), but nowadays, many studies also aim to validate the effects of existing stress through laboratory parameters (objective) [39,40,41]. In 1926, Walter Cannon described the body’s response to stress, known as the “fight or flight” response, in which the adrenal medulla hormone adrenaline is secreted, aiding in fight or flight processes. However, János Selye recognized that catecholamines (including adrenaline) only remain in circulation for a short time, unlike steroid hormones such as cortisol, which can be detected in certain tissues even weeks later [42].

Indeed, early and chronic stress in middle childhood can manifest as dysregulation of the hypothalamic–pituitary–adrenal (HPA) axis, associated with short-term and long-term physical, behavioral, cognitive, and mental problems. While the results of individual studies may not be consistent, generally, it can be said that acute stressors elicit an exaggerated response from the HPA axis, resulting in higher cortisol levels, while chronic stress can lead to a blunted response of the HPA axis, resulting in lower cortisol levels and overstimulation of the axis [41,43,44].

Currently, among the available markers, cortisol is considered the most reliable biomarker for measuring physiological stress levels [40]. However, studies conducted on children may have limitations.

Cortisol is also known as the stress hormone. Nowadays, it is routinely measured in laboratories from blood, saliva, and urine samples. However, these methods are suitable for monitoring the current cortisol level. Samples need to be taken at a specific time of day (for saliva and serum) multiple times to establish a profile, or a time-consuming collection method is required (such as 24 h urine collection). Therefore, these methods are unsuitable for analyzing a population. Twenty-four-hour urine collection only reflects the cortisol level of one day and cannot detect changes that occurred over weeks or months. While saliva and urine cortisol show real-time levels, hair cortisol analysis can serve as a complementary tool for monitoring chronic stress and indicating longer term systemic cortisol concentration. The quantitative measurement of cortisol using this new method is increasingly being applied to identify the effects of stress in various pathological conditions, ranging from chronic pain to acute myocardial infarction. As it enables the long-term, month-to-month measurement of systemic cortisol exposure, hair cortisol analysis can become a useful tool capable of answering clinical questions that previously remained unanswered by other tests [39].

Hormones present in the bloodstream reach the hair follicles and become incorporated into the hair strands. Hormone levels measured in the hair can reflect the average cortisol hormone level over several months. Since hair grows approximately 1 centimeter per month (within the range of 0.6–1.4 cm), a 2–3 centimeter hair sample reflects the average hormone levels from the preceding 2–3 months before the time of sampling. Studies comparing cortisol levels in human hair with cortisol concentrations measured in saliva, serum, and urine of the same individuals have found no discrepancies [45].

As measuring cortisol from hair is a non-invasive procedure, it has become an increasingly popular research topic. Several publications in the literature have addressed the possible measurement methods and compared their accuracy [46,47]. The ultimate goal is to apply this method in clinical settings as a precise and reliable parameter. Hair is easily accessible, samples can be easily stored at room temperature, and cortisol can be measured using relatively simple methods. Hair cortisol measurement has proven to be a valuable diagnostic tool in conditions involving extreme hypercortisolism, such as Cushing’s syndrome. Hair cortisol concentrations have also been associated with metabolic syndrome, obesity, and cardiovascular diseases [48]. Recently, hair cortisol concentration has also been considered a marker of stress in children [49,50].

Our aim is to establish a non-invasive measurement method based on the international literature that could be suitable for objectively assessing chronic stress exposure. Our objective was to explore anxiety and factors influencing anxiety among adolescent students in high school, as well as to understand other factors related to anxiety, such as family characteristics, and psychosomatic symptoms. Additionally, we specifically aimed to examine whether data obtained from different measurement tools for anxiety assessment (psychological tests and biological markers) similarly indicate the level of anxiety.

In our research, we sought to answer the following questions:

(1) How does the level of anxiety change during different periods of the school year (end of the year vs. beginning of the year)?
(2) How do the results of psychological tests and biological sample collection relate to each other?
(3) What are the differentiating factors underlying anxiety?
(4) What is the correlation between anxiety and psychosomatic symptoms?
(5) How does anxiety relate to performance expectations related to school tasks?

Our research started from the following hypotheses:

(1) The level of anxiety decreases significantly after the summer vacation.
(2) We can demonstrate a correlation between the level of anxiety measured by the psychological test and the cortisol level.
(3) Certain characteristics, such as the gender of the respondent, the type of secondary school where he/she studies, the highest education level of the parents, and the family environment influence the level of anxiety.
(4) A higher level of anxiety results in the appearance of psychosomatic symptoms.

## 2. Materials and Methods

Our research was conducted in May 2019 and September 2019 in secondary educational institutions of a county-level city (vocational school, vocational high school, and high school). The questionnaires were completed at two different time points, simultaneous with the biological sample collection. The first data collection took place at the end of the 2018/2019 school year, and the second data collection occurred on the first day of the 2019/2020 school year. The biological samples were processed at the laboratory of the Faculty of Health Sciences, University of Pécs, after the completion of the questionnaires. The entire quantity of samples was utilized during the laboratory procedures.

For the sampling method and procedure for the design of the experiment, we used cluster sampling, where students from the 10th grade of randomly selected schools were chosen as the subjects for the study to ensure comparability with international studies. Regarding the questionnaire survey, the inclusion criterion for randomly selected 10th grade students from the chosen schools was their consent to participate in the research, given by the research subject and their legal guardian. The biological sample collection took place after the completion of the questionnaire survey. Using systematic sampling among the students who filled out the questionnaire, we randomly selected those from whom we planned to determine cortisol levels from their hair samples. Regarding the biological sample collection, in addition to consent for the biological sample collection, the inclusion criterion was a minimum hair length of 3 cm. Before the sampling, we informed the students that only cortisol level measurement would be conducted from their hair samples, which was necessary because various other substances can be detected in hair. Regarding the biological sample collection, those who received steroid treatment in the month preceding the sampling or were undergoing treatment at the time of sampling were excluded.

The biological sample collection took place on two occasions: the first one was at the end of May while the school year was still ongoing. Therefore, the cortisol levels in the hair reflect the average values for the school months of March, April, and May. The second sample collection took place in the first week of September to examine the cortisol levels corresponding to the preceding three-month summer break. The students filled out the same questionnaire on both occasions, and demographic data were repeated during the second occasion only if there were any changes in the research subject’s life in the three months prior to the questionnaire administration. At the beginning of the study, voluntary participants were assigned a code that ensured their anonymity. It also facilitated tracking of participating students, matching of pre-test and post-test data, and linking the cortisol content of hair samples from the two different time points. Only the research leaders had access to the codes, and the code list was destroyed after the second sample collection. In reporting the results of the study, the schools are not identified by name, only the types of schools are mentioned.

Sample size: The minimum sample size determined by G*Power statistical power analysis was 115 people (to compute statistical power analyses). Due to the biological sampling carried out at two different times during the research, we did not aim for a high number of elements. The study was voluntary, a total of 137 people took part in the research, and in the end, we were able to evaluate the answers of 125 people [51].

A total of 125 participants took part in the questionnaire survey, and 100 participants provided hair samples. According to the literature, the willingness to provide hair samples may vary among young individuals.

### 2.1. Data Collection Methods

The measurement instrument used in the questionnaire survey consisted of self-designed questions (mainly related to sociodemographic characteristics) and standardized measurement tools. The standardized tools included the Spielberg State–Trait Anxiety Inventory, the Clear Communication Subscale of the Family Dynamics Measure-II questionnaire, and the Multiple Social Perceived Support Scale, as well as questions related to psychosomatic symptoms from the Health Behavior in School-Aged Children (HBSC) questionnaire. [52,53,54,55] The Spielberg State–Trait Anxiety Inventory measures the intensity of anxiety through 20 statements. The Clear Communication Subscale of the Family Dynamics Measure-II [53] examines family communication along the following statements, using a 6-point Likert scale: in my family, (1) we discuss important things with each other; (2) someone listens when I speak; (3) when we do not understand each other, we ask questions; (4) if we misunderstand something, we talk about it until it is clarified. The level of support received from the family was measured using the Family Subscale of the Multiple Social Perceived Support Scale [55] (MSPSS), which examines the perceived level of family support through four items. The statements include: (1) my family really tries to help me; (2) I receive the emotional support and care I need from my family; (3) I can talk to my family about my problems; (4) my family is willing to help me make decisions.

#### The Process of Generating the Scales Included in the Data Analysis

For the analysis of the Spielberg State–Trait Anxiety Inventory, after recoding the reversed items, we examined the reliability of the questionnaires. Subsequently, we calculated the scale scores for each statement by summing the assigned values, resulting in the creation of state and trait anxiety scales for both the May and September data collection. The Family Dynamics Measure-II Clear Communication Scale [53] was generated by summing the scores assigned to each statement. The creation of the Multiple Social Perceived Support Scale [55] followed the same process as the Clear Communication Scale. Regarding the psychosomatic symptoms commonly observed during adolescence, our questionnaire asked about the frequency of occurrence of the following symptoms, following the approach of the HBSC research [54] (1) headache, (2) stomach aches and abdominal pain, (3) back pain, (4) low mood, (5) irritability, (6) fear, nervousness, (7) insomnia, (8) waking up during the night, (9) dizziness, (10) fatigue, exhaustion, (11) nausea, vomiting. To capture the overall occurrence of psychosomatic symptoms, we created an aggregated index by summing the scores assigned to the frequency of occurrence for each symptom (5 = almost every day; 4 = several times a week; 3 = once a week; 2 = once a month; 1 = less frequently or never). Accordingly, a higher score indicates a higher occurrence of psychosomatic symptoms. The reliability indices of the applied measurement instruments are presented in the first table (Table 1).

The reliability indicators of the scales used are high; therefore, each of them proved to be suitable for analysis.

A Kolmogorov–Smirnov test has been used to assess the normality of the data distribution for each scale. If the data were normally distributed, parametric tests such as paired *t*-tests, independent *t*-tests, ANOVA, and Pearson correlation were employed for the analysis.

### 2.2. The Process of Biological Sample Collection

From those subjects who consented to participate in the study, we collected approximately 10–20 mg of hair using scissors. The hair should be collected from the posterior vertex of the head, as this area exhibits the highest hair growth rate (1 cm/1 month) and the least individual variability in cortisol [45]. This means that cortisol concentration measurement is based on the average hair growth rate of 1 cm/month during the three-month period prior to sample collection [56]. This amount corresponds to approximately 100–120 hair strands. The last 3 cm of each hair strand were placed in pre-labeled Eppendorf tubes. Cortisol concentrations were determined from this 3 cm hair segment. The samples were processed and can be stored for up to 12 months at room temperature [57]. The hair samples were prepared for measurement according to the method described by Greff and colleagues [58]. The samples were accurately weighed on an analytical balance, and then finely chopped using surgical scissors. The chopped samples were washed in 1 mL of isopropanol for 2 × 3 min at room temperature, and the supernatant was discarded as it was not used. Afterward, the samples were air-dried.

**Figure 1:**
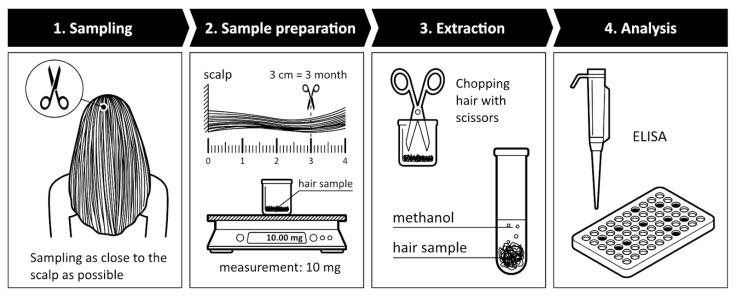
Process of hair sample preparation and measurement.

The next step is extraction. The samples were incubated in 1 mL of methanol for 16 h (200 rpm, 52 °C). The supernatant was transferred to an Eppendorf tube, and then 1 mL of acetone was added and incubated at room temperature (200 rpm) for 5 min. The supernatant was removed. This process was repeated once. Next, the samples were evaporated using nitrogen, followed by measurement. For measurement, the samples were dissolved in PBS buffer: 10 mg of sample + 75 microliters of PBS. The measurement was performed using a modified cortisol saliva ELISA kit, following the instructions provided in the cortisol kit (Cortisol Saliva Kit REF: DSNOV20, LOT: S-CORT-5240A NovaTec Immundiagnostica GMBH, Dietzenbach, Germany). The instrument used was an ELISA Microplate Reader: Type: 357, REF: 51119100, SN: 357-906094T (ThermoFisher, Waltham, MA, USA) (Figure 1).

For cortisol levels, since the Kolmogorov–Smirnov test yielded a *p*-value below 0.05, non-parametric tests (Wilcoxon test, Mann–Whitney test, Kruskal–Wallis test, Spearman correlation) were used for the statistical analyses related to cortisol levels.

## 3. Results

### 3.1. The Main Characteristic of Sample

In our follow-up investigation, a total of 125 16–17-year-old high school students were involved. During the first data collection, they filled out the questionnaire, and among them, those who also underwent biological sampling were selected. In the second data collection, a total of 87 students completed the questionnaire. During the first data collection, 74 boys (59.2%) and 51 girls (40.8%) participated in the study. A total of 56.8% (*n* = 71) of the interviewed high school students live in cities, most of them (69.6%) with both parents. Of them, 23.2% attended high school, 48.8% attended vocational high school, and 28% attended vocational school. Regarding the highest educational attainment of parents, among fathers, those with a vocational certificate are in the majority (41.6%), while only 18.4% completed college or university studies. Among mothers, the proportion of those with a diploma is higher (27.2%), while the proportion of those with a vocational qualification is lower (21.5%). For further analysis, we divided both fathers and mothers into three groups based on their highest educational attainment. Accordingly, individuals with primary school education and vocational qualifications were grouped into the category of lower educational attainment at the secondary school level. Those who graduated from secondary school and obtained certificates through secondary education-based vocational training were grouped into the category of those with a secondary school diploma. Individuals with a college or university degree were grouped into the category of higher education attainment; this section may be divided by subheadings. It should provide a concise and precise description of the experimental results, their interpretation, as well as the experimental conclusions that can be drawn.

### 3.2. Results of the Study on Anxiety and Differentiating Factors of Anxiety

In our study, we used the State–Trait Anxiety Inventory (STAI) by Spielberg to measure anxiety at two different time points. Following the completion of the questionnaire, we conducted biological sampling to measure cortisol levels in hair. For boys, both state and trait anxiety showed levels of anxiety in the first data collection that were similar to the standard values (Table 2), while the level of anxiety significantly decreased during the second data collection.

Among girls, both state and trait anxiety showed higher values compared to the standard at both data collection time points. The analysis of differentiating factors of anxiety (Table 3) revealed significant differences among boys and girls (May STAI-S: t = −4.00, *p* < 0.01; May STAI-T: t = −7.58, *p* < 0.01; September STAI-S: t = −5.89, *p* < 0.01; September STAI-T: t = −7.82, *p* < 0.01), as well as among different types of schools (May STAI-S: F = 3.44, *p* < 0.001; May STAI-T: F = 8.60, *p* < 0.001; September STAI-S: F = 8.63, *p* < 0.001; September STAI-T: F = 11.76, p<0.001) in terms of both state and trait anxiety. The statistical tests highlighted higher stress levels among girls and students in high schools. Cortisol levels were significantly higher in boys (Z = −4.07, *p* < 0.001) and students in vocational high schools (χ^2^ = 26.17, *p* < 0.001) during the first measurement, while the differences between boys and girls disappeared during the second measurement (Z = −0.46, *p* = 0.64), with only the differentiating effect of school type remaining (χ^2^ = 11.02, *p* = 0.004). Examining the highest educational attainment of parents, the father’s highest educational level did not prove to be a differentiating factor, while the mother’s highest educational level had a significant impact on the child’s trait anxiety during the May data collection (F = 3.10, *p* = 0.49). State and trait anxiety were highest among children of mothers with a higher education level in both measurement time points. The mother’s highest educational level influenced cortisol levels during the first measurement in May, with children of mothers with higher education levels having lower cortisol levels.

One of the most important questions in our research was whether a significant change in anxiety levels among high school students could be detected between the two measurement time points. We aligned the two measurement time points with the end of the school year and the beginning of the following school year, assuming that during the nearly three-month summer break, adolescents would start the new school year with lower levels of anxiety, free from the weight of school tasks. All three measures confirmed a significant change in anxiety levels. However, while state anxiety (t = 3.51, *p* = 0.001) and trait anxiety (t = 3.75, *p* < 0.001) showed a decrease in anxiety levels, cortisol levels increased (Z = −2.58, *p* = 0.01) in the examined hair samples (Table 4).

We hypothesized that anxiety among high school students may be related to school tasks, which could explain why anxiety levels are lower at the end of the summer break and the beginning of the school year. In investigating performance anxiety related to school tasks, we asked the participating students how burdensome they perceived school tasks to be and compared their responses to the level of anxiety. Our results showed significant differences in both measurement time points regarding both state anxiety (May: F = 9.31, *p* < 0.001; September: F = 6.54, *p* = 0.006) and trait anxiety (May: F = 6.81, *p* < 0.001; September: F = 6.88, *p* < 0.001) based on the expectations associated with school tasks. According to the data in Table 5, it becomes evident that the more burdensome the school tasks are perceived, the higher the level of anxiety experienced. During the May data collection, we did not find a significant difference in cortisol levels based on school performance anxiety (χ^2^ = 4.72, *p* = 0.19), while the September measurement showed a significant difference (χ^2^ = 8.93, *p* = 0.03).

During the examination of psychosomatic symptoms, we inquired about the occurrence and frequency of various symptoms present in the HBSC survey. Among the examined psychosomatic symptoms, there is a notable presence of apathy, irritability, and fatigue/exhaustion, which not only affect the participating adolescents the most but also pose the greatest problem in terms of frequency. As a first step, we examined the correlation between these symptoms and the level of anxiety. It can be observed that both apathy (Table 6), irritability (Table 7), and fatigue/exhaustion (Table 8) show a positive correlation with the intensity and frequency of the symptoms. The cortisol level only showed a correlation with apathy during the first measurement.

Analyzing the relationship between psychosomatic symptoms and anxiety further, we created an aggregated index from the psychosomatic symptoms as described in Section 2 and examined the correlations between variables. Both state anxiety (May: r = 0.70, *p* < 0.001; September: r = 0.55, *p* < 0.001) and trait anxiety (May: r = 0.74, *p* < 0.001; September: r = 0.66, *p* < 0.001) showed a significant positive moderate association with psychosomatic symptoms. This indicates that the stronger the student’s anxiety, the greater the presence and intensity of psychosomatic symptoms in their life (Table 9).

When examining gender differences, both the May and September data collection revealed significant differences in psychosomatic symptoms between boys and girls (May: F = 0.51, *p* < 0.001; September: F = 0.12, *p* < 0.001). Both data collections indicated a higher prevalence of symptoms among girls (May: Mean = 32.81 vs. 24.90; September: mean = 27.36 vs. 19.47). Regarding school types, the Kruskal–Wallis test showed significant differences in both the May (χ^2^ = 13.46, *p* = 0.001) and September (χ^2^ = 26.49, *p* < 0.001) data collections. The examination of psychosomatic symptoms directs attention to students attending high schools.

Considering that we are dealing with adolescent students, the examination of anxiety cannot overlook the influence of family factors, which strongly impact adolescents’ well-being, overall mood, and, not least, their ability to cope with stressful situations. In the case of family variables, alongside the parents’ highest level of education, we examined the characteristics of family communication and perceived support from the family during the first data collection using the four-item subscale of Clear Communication from the Family Dynamics Measure-II and the Multiple Social Perceived Support Scale (Table 10).

The Clear Communication Scale shows a weak, negative, and statistically significant relationship with both state anxiety (*r* = −0.29, *p* = 0.001) and trait anxiety (*r* = −0.22, *p* = 0.01), as well as with psychosomatic symptoms (*r* = −0.29, *p* = 0.001). As for the Multiple Social Perceived Support Scale, the correlations are slightly stronger. Here, too, there are negative, weak to moderate, and significant relationships between perceived support and anxiety (STAI-S: *r* = −0.32, *p* < 0.001; STAI-T: *r* = −0.29, *p* = 0.001), as well as between perceived support and psychosomatic symptoms (*r* = −0.32, *p* < 0.001). This means that the lack of family communication and low levels of perceived support significantly increase the level of anxiety and the frequency of psychosomatic symptoms.

## 4. Discussion

Our study measured the stress levels of high school students and drew conclusions about the factors that contribute to increased levels of anxiety. The research examined trait and state anxiety values subjectively at two time points using a standardized questionnaire. The first time point was in May 2019, at the end of the school year, and the second time point was in early September 2019, at the beginning of the following school year. Gender differences as differentiating factors were evident in both measurement occasions. For boys, the level of anxiety approached international standard values for both types of anxiety, while for girls, these values were higher. The obtained anxiety scores clearly indicated higher stress exposure among girls. This difference can be explained by adolescent changes that pose hormonal and mental burdens on high school students, especially girls, making it more difficult for them to adapt. Socially sensitive girls struggle more with the obstacles they face, as indicated by their self-reported anxiety scores. Significant differences were confirmed between the anxiety scores measured at the end of the school year and those measured at the beginning of the subsequent year. Both genders responded to the school break with a clear decrease in anxiety, suggesting that the absence of school attendance as a stress factor had a positive impact on their mental state. This can be explained by the summer break, rest, and a period free from responsibilities, during which they encountered fewer stressors. Boys experienced a more significant decrease in anxiety levels. It is likely that more impulsive and less mature teenage boys find it easier to let go and forget about past matters or upcoming tasks.

### 4.1. Questionnaire Survey and Cortisol Level

Contrary to the results of the questionnaire survey, the cortisol values do not reflect the difference between the two measurement time points. Our intention was to support the results of the questionnaire survey with this objective measurement opportunity, but instead of a decrease in cortisol levels, we encountered an unexpected increase. Therefore, hair samples indicated higher stress exposure during the summer period compared to the last weeks filled with tasks associated with the end of the school year in May. It is thought-provoking that subjective tests measuring the level of anxiety show a decrease in anxiety, whereas objective indicators inform us of the opposite. The quantity of cortisol levels in individuals can vary greatly, and the physiological threshold within which our measurements fall is quite wide. Cortisol levels can be influenced by numerous factors, including psychological and mental characteristics that develop during childhood due to parental influences, as well as the effectiveness of coping strategies during adolescence. These factors shape an individual’s stress tolerance, which ultimately manifests in cortisol levels. However, our study did not extend to examining these factors. Differences between the level of anxiety measured by psychological tests and cortisol levels obtained through biological sampling have also been supported by other previous studies [58], thus confirming the results of Lu et al. [59]. According to their study, there is a positive correlation between the perception of depressive symptoms and cumulative hair cortisol levels in boys. Higher levels of anxiety symptoms are associated with lower hair cortisol concentration only in girls. The mentioned researchers’ study provided the first evidence that depressive symptoms are associated with increasing hair cortisol concentration in boys, while anxiety symptoms are associated with HPA axis hypoactivity in girls, which reduces cortisol concentration.

### 4.2. Differences between School Types

Since the age group under study participates in different types of education at the secondary level, we considered it important to examine the level of anxiety based on school types. By analyzing data representing acute and general states, we obtained results. Consistent with our preliminary assumption, the school type showed the expected pattern. Accordingly, students attending high schools, which have higher expectations, experienced significantly greater burdens, which they themselves perceive, compared to children attending schools with lower requirements. Vocational schools provide professional training, while vocational high schools represent intermediate level education, and high schools represent the highest level of secondary education. Acquiring usable knowledge is a fundamental expectation for students everywhere. However, vocational school students receive significantly less theoretical education and undergo fewer assessments compared to their peers in the other two school types. With the introduction of dual education, vocational school students have been freed from strict school frameworks and spend 50% of their time acquiring their learned profession in practical settings. This type of education, combining school and the world of work, provides more opportunities for relaxation, conversation, stress relief, or simply engaging in carefree activities, which can be an effective means of coping with stress. The anxiety scores reflect all of these factors well. We detected higher levels of anxiety in school types with higher requirements. Students attending vocational schools experienced the least anxiety, followed by vocational high school students, while the highest scores were observed among students in high schools.

Among the two measurement time points, the smallest decrease in anxiety was observed among students in high schools, indicating that the summer break was the least restful and least anxiety-free for them, as they were unable to escape the burdensome concerns even during the summer vacation. On the other hand, the anxiety of adolescents attending vocational high schools and vocational schools decreased significantly during the examined period (Table 3). The anxiety of students in vocational schools decreased to the greatest extent, which supports our previous assertion regarding the orderliness, strictness, and “better livability” of their school environment.

### 4.3. Parental Expectations

The analysis concludes that there was no significant relationship found between a child’s anxiety level and the father’s highest level of education. However, for mothers, it was observed that higher maternal education was significantly associated with higher levels of anxiety in adolescents. Since our study did not include more in-depth questions regarding the mother’s highest level of education and anxiety levels, we can rely on previous educational sociology research [60] which directs attention to the role of maternal education in a child’s school life. Additionally, considering the adolescent age group and the influence of family dynamics, family communication was examined using the CCS scale, and the level of perceived support within the family was assessed using the MSPSS scale. Both scales showed a negative, weak to moderate correlation with both state and trait anxiety levels. This means that effective communication and a high level of perceived support reduce anxiety in both dimensions.

In addition to parental expectations, communication climate, and family support, it is important to consider how students relate to school tasks and how burdensome they perceive them to be. Although this assessment is inherently subjective and influenced by numerous factors that were not examined in this study, our results indicate that the more students perceive school tasks as burdensome, the higher levels of anxiety they experience in both state anxiety and trait anxiety dimensions. These findings are supported by both the May and September data (Table 5).

### 4.4. Psychosomatic Symptoms

The negative effects of improperly managed stress have been well-known since the work of János Selye. The presence of chronic “bad” stress also entails somatic manifestations, which may start with minor complaints but can become a source of numerous serious problems if left untreated or improperly addressed. Previous studies have drawn attention to the emergence of psychosomatic symptoms related to school tasks and performance anxiety [61,62]. Among these symptoms, headaches, backaches, and gastrointestinal complaints are noteworthy, as each of them can negatively affect performance. Therefore, it is important to address them specifically. Anxiety does not always manifest as physical symptoms; it can also manifest as mental problems, which were more observable among the students involved in the study than physical complaints. The highest rates were found for apathy, irritability, and fatigue, all of which showed a correlation with higher levels of anxiety (Table 6, Table 7 and Table 8). The aggregated index formed from psychosomatic symptoms encompasses both the number of symptoms and their frequency of occurrence (Table 9). This index drew attention to the greater involvement of girls and a strong correlation with the level of anxiety.

### 4.5. Limitations

The limitations of our research primarily include the small sample size and attrition of participants during the follow-up assessment, particularly evident in the biological sampling where many boys cut their hair over the summer, requiring us to exclude them from the second sampling. In our study, we focused only on the clear communication scale and the Multiple Social Perceived Support Scale among the family characteristics, without examining other family factors (home atmosphere, social circumstances). Therefore, we did not aim to explore the underlying family factors contributing to anxiety in greater depth. In future investigations of stress among high school students, it will be crucial to pay greater attention to a larger sample size and conducting a detailed exploration of family characteristics.

## 5. Conclusions

The significance of studying stress, anxiety, and related phenomena, including the widely experienced psychosomatic symptoms, is undeniable. It is especially important to focus on the age group that struggles with anxiety stemming from parental and school expectations amidst the changes in adolescence. The aim of our study was to identify the differentiating factors of anxiety and explore the factors associated with anxiety and a key objective was to compare the levels of self-reported anxiety measured by psychological instruments with the measurable cortisol levels extracted from hair samples.

We did not find a positive correlation between perceived stress (subjective measurement) and cortisol levels isolated from hair samples (objective indicator). The correlation between perceived stress and psychosomatic symptoms suggests that subjective tests used to measure anxiety provide a more accurate indication of anxiety levels in the examined age group compared to cortisol measurements from hair samples.

Our findings confirm previous studies showing a decrease in cortisol levels accompanying an increase in anxiety levels among girls, and this inverse relationship is also observed in boys. Furthermore, our data supplement previous research by demonstrating this relationship not only in the 10–12 age group but also among 16–17-year-olds. Answering the question of which biological markers can reliably indicate chronic stress exposure may prompt further investigations.

## Figures and Tables

**Table 1:** Reliability indicators.

Measuring Tool	Number of Items	Cronbach’s Alpha
Measure: State anxiety (STAI-S)	20	0.92
2. Measure: Trait anxiety (STAI-T)	20	0.88
3. Measure: State anxiety (STAI-S)	20	0.93
4. Measure: Trait anxiety (STAI-T)	20	0.92
5. Measure: Psychosomatic symptoms	11	0.85
6. Measure: Psychosomatic symptoms	11	0.91
Clear Communication Scale	4	0.79
Multiple Social Perceived Support Scale	4	0.89

**Table 2:** The STAI standard values. (Source: Sipos and Sipos, 1988 [52]).

Genders	STAI-S Standard Values	STAI-T Standard Values
Mean (Standard Deviation)		
Male	38.40 (10.66)	40.96 (7.78)
Female	42.64 (10.79)	45.37 (7.97)

**Table 3:** Differentiating aspects of anxiety.

	STAI-S 1.	STAI-S 2.	STAI-T 1.	STAI-T 2	Cortisol 1	Cortisol 2
Mean (Standard Deviation)						
Genders						
Male	38.45 (9.46)	38.45 (9.46)	38.45 (9.46)	38.45 (9.46)	38.45 (9.46)	38.45 (9.46)
Female	46.04 (11.32)	46.04 (11.32)	46.04 (11.32)	46.04 (11.32)	46.04 (11.32)	46.04 (11.32)
	t = −4.00; *p* < 0.01	t = −4.00; *p* < 0.01	t = −4.00; *p* < 0.01	t = −4.00; *p* < 0.01	t = −4.00; *p* < 0.01	t = −4.00; *p* < 0.01
School type						
High school	44.78 (11.17)	43.10 (11.07)	48. 51 (9.14)	46.07 (8.29)	1.06 (0.85)	2.64 (3.15)
Vocational high school	41.93 (11.13)	37.84 (12.01)	44.30 (9.43)	41.21 (11.49)	2.03 (2.59)	4.60 (3.72)
Vocational school	37.79 (9.15)	30.42 (6.05)	39.00 (8.11)	33.14 (4.77)	3.50 (2.14)	3.27 (1.85)
	F = 3.44; *p* < 0.001	F = 8.63; *p* < 0.001	F = 8.60; *p* < 0.001	F = 11.76; *p* < 0.001	χ^2^ = 26.17; *p* < 0.001	χ^2^ = 11.02; *p* = 0.004
Mother’s education level						
Under degree	39.70 (10.78)	33.75 (10.85)	41.36 (9.74)	38.30 (9.07)	3.54 (4.07)	3.12 (3.12)
High school degree	41.14 (11.54)	39.42 (11.65)	43.61 (9.69)	41.57 (10.92)	1.99 (0.91)	3.65 (3.5)
University degree	44.65 (10.31)	39.66 (11.06)	47.21 (8.53)	42.29 (10.09)	1.61 (0.80)	3.75 (2.99)
	F = 1.72; *p* = 0.18	F = 1.96; *p* = 0.14	F = 3.10; *p* = 0.49	F = 0.96; *p* = 0.38	χ^2^ = 7.18; *p* = 0.02	χ^2^ = 1.14; *p* = 0.56
Complete sample	41.43 (10.84)	37.74 (11.47)	43.75 (9.57)	40.82 (10.35)	2.35 (2.31)	3.56 (3.30)

**Table 4:** Anxiety data to cortisol levels comparison.

	STAI-S II. Data Collection	STAI-T II. Data Collection	Cortisol 2. Measurement
STAI-S I. data collection	*t* = 3.51; *p* = 0.001		
STAI-T I. data collection		*t* = 3.75; *p* < 0.001	
Cortisol 1. measurement			Z = −2.58; *p* = 0.01

**Table 5:** How much do school tasks burden you?

	STAI-S 1.	STAI-S 2.	STAI-T 1.	STAI-T 2.	Cortisol 1	Cortisol 2
Mean (Standard Deviation)						
They are not distressing	35.37 (8.10)	29.56 (6.17)	38.18 (9.67)	33.87 (9.50)	2.51 (1.74)	7.83 (6.10)
They are a bit distressing	39.10 (8.87)	36.82 (11.70)	42.36 (9.13)	40.25 (10.61)	2.62 (2.90)	2.32 (1.90)
Quite distressing	44.43 (10.90)	41.80 (10.07)	45.97 (8.25)	43.11 (7.46)	1.93 (1.79)	2.92 (2.61)
Very distressing	54.22 (13.77)	48.00 (12.94)	53.44 (9.13)	53.16 (8.99)	2.13 (0.87)	2.68 (1.64)
	F = 9.31; *p* < 0.001	F = 6.54; *p* = 0.006	F = 6.81; *p* < 0.001	F = 6.88; *p* < 0.001	χ^2^ = 4.72; *p* = 0.19	χ^2^ = 8.93; *p* = 0.03

**Table 6:** Apathy.

	STAI-S 1.	STAI-S 2.	STAI-T 1.	STAI-T 2.	Cortisol 1	Cortisol 2
Mean (Standard Deviation)						
Almost daily	52.86 (10.47)	48.50 (.6.45)	52.20 (6.42)	54.50 (7.72)	2.03 (0.76)	1.39 (0.78)
Several times a week	46.33 (9.99)	46.50 (10.43)	49.82 (8.10)	51.71 (9.26)	1.55 (0.59)	4.57 (3.19)
Weekly	39.48 (8.03)	38.17 (7.47)	41.92 (9.00)	41.70 (8.98)	2.39 (2.02)	3.91 (4.74)
Per month	35.40 (7.19)	38.81 (12.76)	38.06 (5.79)	40.14 (6.66)	2.61 (1.84)	3.16 (3.53)
Rarely or never	31.60 (8.99)	29.68 (7.78)	33.10 (5.19)	32.68 (7.58)	5.17 (6.19)	2.14 (1.47)
	F = 16.15; *p* < 0.001	F = 8.06; *p* < 0.001	F = 21.15; *p* < 0.001	F = 16.37; *p* < 0.001	χ^2^ = 12.71; *p* = 0.01	χ^2^ = 5.41; *p* = 0.24

**Table 7:** Irritability.

	STAI-S 1.	STAI-S 2.	STAI-T 1.	STAI-T 2.
Mean (Standard Deviation)				
Almost daily	45.30 (12.15)	51.50 (15.60)	48.50 (9.65)	56.50 (7.04)
Several times a week	46.16 (11.41)	41.76 (8.43)	48.54 (9.54)	45.05 (11.81)
Weekly	41.13 (8.88)	39.07 (10.75)	42.89 (8.06)	43.21 (8.83)
Per month	34.40 (8.58)	40.60 (11.82)	39.31 (8.08)	42.44 (7.32)
Rarely or never	38.35 (9.28)	29.85 (7.96)	38.15 (8.00)	33.11 (7.57)
	F = 5.63; *p* < 0.001	F = 7.19; *p* < 0.001	F = 7.25; *p* < 0.001	F = 10.09; *p* < 0.001

**Table 8:** Tired, exhausted.

	STAI-S 1.	STAI-S 2.	STAI-T 1.	STAI-T 2.
Mean (Standard Deviation)				
Almost daily	45.42 (12.08)	41.11 (10.92)	48.46 (8.45)	45.38 (10.47)
Several times a week	43.72 (10.26)	43.26 (11.81)	45.84 (9.25)	46.40 (9.17)
Weekly	40.96 (7.62)	42.41 (10.70)	42.28 (8.74)	43.58 (10.01)
Per month	31.31 (5.53)	37.36 (14.10)	34.37 (5.51)	38.81 (9.17)
Rarely or never	31.80 (12.07)	29.34 (5.07)	35.40 (7.47)	33.50 (7.25)
	F = 7.30; *p* < 0.001	F = 6.95; *p* < 0.001	F = 9.44; *p* < 0.001	F = 7.26; *p* < 0.001

**Table 9:** Correlation between psychosomatic symptoms and anxiety.

	STAI-S 1.	STAI-S 2.	STAI-T 1.	STAI-T 2.
Psychosomatic symptoms (aggregate variable)	r = 0.70 *p* < 0.001	r = 0.55 *p* < 0.001	r = 0.74 *p* < 0.001	r = 0.66 *p* < 0.001

**Table 10:** Correlation between family characteristics and anxiety.

	STAI-S 1.	STAI-T 1.	Psychosomatic Symptoms (Aggregate Variable)
Clear Communication Scale	*r* = −0.29; *p* = 0.001	*r* = −0.22; *p* = 0.01	*r* = −0.29; *p* = 0.001
Multiple Social Perceived Support Scale	*r* = −0.32; *p* < 0.001	*r* = −0.29; *p* = 0.001	*r* = −0.32; *p* < 0.001

## Data Availability

The data of the research are available from G.P.S. and T.S.-R.
